# Artificial intelligence in oncology: empowering clinicians for responsible integration

**DOI:** 10.1016/j.esmorw.2026.100737

**Published:** 2026-07-14

**Authors:** E. Colliver, E. Parisini, B. Arefaine, PIVOT team, C.R.S. Banerji, G.E. Verghese, A. Grigoriadis

**Affiliations:** 1 PharosAI, School of Cancer & Pharmaceutical Sciences, Faculty of Life Sciences and Medicine, King’s College London, London, UK; 2 The Alan Turing Institute, London, UK; 3 University College London NHS Trust, London, UK; 4 Cancer Bioinformatics, School of Cancer & Pharmaceutical Sciences, Faculty of Life Sciences and Medicine, King’s College London, London, UK; 5 Breast Cancer Now Research Unit, School of Cancer and Pharmaceutical Sciences, Faculty of Life Sciences and Medicine, King’s College London, London, UK

**Keywords:** AI education, AI interpretability, AI regulation, clinical AI, clinical decision support systems, federated learning

## Abstract

Artificial intelligence (AI) is rapidly reshaping oncology, from diagnosis to treatment planning and clinical research. This perspective defines the oncologist in the era of AI as a clinician able to critically interpret, supervise, and communicate AI outputs, while understanding the principles, limitations, and ethical implications of these tools. We ground this discussion in a survey of 475 UK-based participants, including cancer patients and survivors, members of the public and healthcare staff. Acceptance of AI was substantial but conditional: it increased sharply with self-reported understanding and depended on assurances of clinician involvement, transparency and data security. These findings motivate the three concerns around which we structure the perspective: ‘AI will replace the clinician’, ‘AI may be biased and unfair’, and ‘AI does not safeguard data’. Using the example of an AI system for cancer treatment recommendation, we illustrate how these concerns can be addressed through practical, technically grounded approaches, including concept-based modelling, uncertainty quantification, and federated learning. Finally, we argue that AI skills development must become an integral part of oncology education, enabling oncologists not only to use AI safely, but also to explain, contextualise, and critically shape its integration into patient-centred cancer care.

## Highlights

• AI is reshaping oncology. Oncologists must feel equipped to understand key ethical and performance considerations.
• Emerging methods aim to enhance the transparency, fairness, and security of AI systems, facilitating clinical adoption.
• Oncologists should be actively involved in shaping AI regulation in clinical cancer care.
• Integrating AI education into oncology curricula will be essential to prepare future clinicians and researchers.
• Any deployment of AI in oncology must uphold and advance the principles of patient-centred care.

## Introduction

Excitement is burgeoning around the opportunities afforded by artificial intelligence (AI) across the healthcare sector. As of early 2026, the US Food and Drug Administration (FDA) listed over 1400 AI-enabled medical devices authorised for marketing in the United States alone. The convergence of AI and oncology has the potential to reshape the clinical, diagnostic, and research paradigms of cancer care. As AI-driven tools are set to increasingly permeate workflows—from histopathological image analysis to predictive modelling of treatment responses—the oncologist’s role is evolving to an informed day-to-day collaborator with AI. This perspective explores what it means to be an oncologist in the era of AI: not merely one who uses AI, but one who understands its principles, limitations, and potential to augment clinical decision making. We examine the interpretative, ethical, and educational frameworks necessary to equip oncologists with the skills and confidence to critically engage with AI technologies, ensuring that innovation translates into improved patient outcomes. Crucially, whether AI improves outcomes will depend not on technical performance alone, but also on patients’ understanding of, and trust in, these tools.

The concerns surrounding trustworthy clinical AI have been extensively discussed in ethical guidance and reporting or evaluation frameworks such as WHO guidance,^1^ CONSORT-AI,^2^ SPIRIT-AI,^3^ DECIDE-AI,^4^ TRIPOD+AI,^5^ and the FUTURE-AI^6^ consensus guideline. Our aim is not to provide a comprehensive review of these implementation challenges, but to translate selected, well-established principles into practical concepts that oncologists may need to understand when evaluating, communicating, or supervising AI tools in cancer care.

### Use cases of AI in oncology

Given the rapid advancement of AI technologies, oncologists, like the broader healthcare workforce, should be enabled to understand, use, and critically evaluate emerging tools, while communicating their outputs clearly to patients. This is increasingly urgent as patients turn to resources such as ChatGPT for guidance on symptoms and treatment options, and as clinicians themselves begin to integrate AI systems into practice.^7^^,^^8^ As these tools enter clinical workflows, oncologists who remain informed about their capabilities and limitations will be best positioned to use them responsibly and explain their role in patient care.

AI is already present in cancer pathways, although adoption remains task- and region-specific rather than ubiquitous. Current use has largely focused on narrow diagnostic tasks in screening, pathology, radiology, and endoscopy, rather than on fully integrated oncology decision making. Examples include the NHS EDITH breast screening trial, NICE’s conditional approval of DERM for skin cancer triage, real-time systems such as GI Genius for colorectal adenoma detection, and FDA-approved or deployed prostate pathology tools such as Paige Prostate Detect and Ibex.^9^, ^10^, ^11^, ^12^, ^13^, ^14^ Together, these examples show growing momentum toward clinical AI integration, while also highlighting that most approved tools remain focused on specific tasks and single data types.

**Figure 1:**
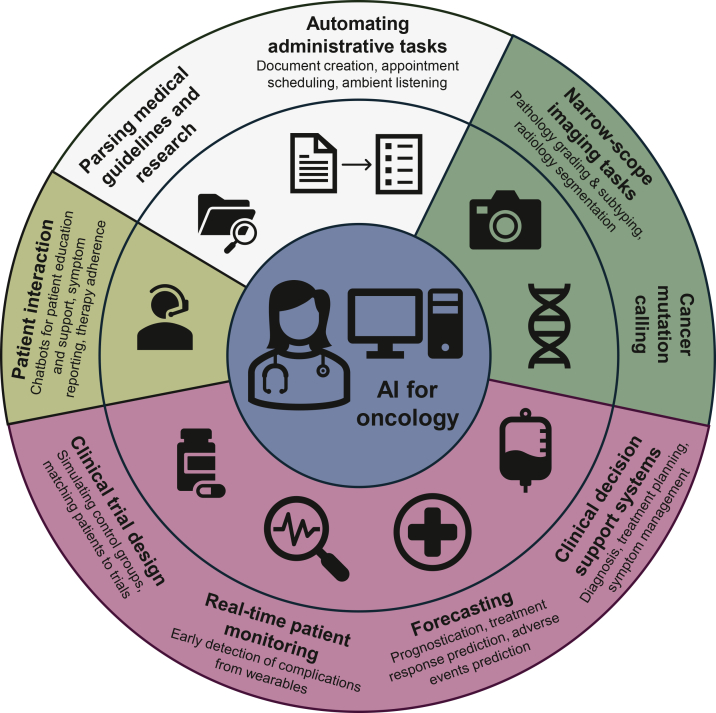
**Use cases of AI in oncology.** AI has the potential to transform oncology through a diverse range of applications that span the clinical, research, and operational continuum. Each colour denotes a major area of impact: pink highlights direct clinical applications; light green captures patient-facing tools; dark green represents data-intensive diagnostic and molecular tasks, from medical image analysis to mutation calling; and gray refers to administrative and research-enabling functions. AI, artificial intelligence.

Looking ahead, AI is likely to support a broader range of oncological practice (Figure 1), including tumour grading, staging, biomarker quantification, treatment recommendation, recurrence and toxicity prediction, patient monitoring through wearables, and patient-facing conversational tools for education, symptom tracking, and adherence support.^15^, ^16^, ^17^, ^18^, ^19^ More advanced ‘agentic’ systems may eventually support parts of the patient pathway by analysing longitudinal multimodal data in the context of medical guidelines, under clinician supervision.^17^ AI is also expected to influence clinical research, including trial recruitment, simulated control arms, drug discovery, and biomarker identification,^20^ while ‘ambient AI’ may reduce administrative burden by supporting documentation, coding, scheduling, resource allocation, and literature synthesis.^21^^,^^22^

This breadth of potential applications underlines the need for oncology-specific standards as AI products enter the clinic. Recent European Society for Medical Oncology (ESMO) initiatives include the ESMO Basic Requirements for AI-based Biomarkers in Oncology (EBAI), which classifies AI-based biomarkers and defines minimum evidence requirements for clinical adoption, and the ESMO Guidance on the Use of Large Language Models in Clinical Practice (ELCAP), which categorises large language model (LLM) applications and provides practical guidance for safe use in oncology.^23^^,^^24^

### Patient trust and the need for AI literacy in oncology

**Figure 2:**
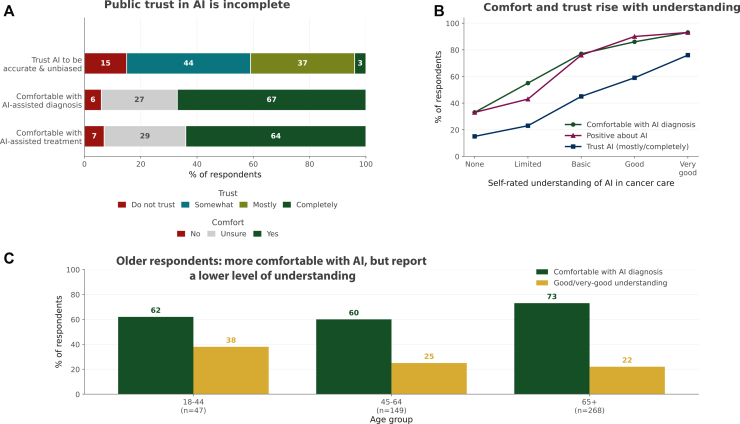
**Patient and public attitudes towards AI in cancer care.** Results from an online survey of 475 UK-based cancer patients and survivors, members of the public, and healthcare staff. (A) Trust in AI, and comfort with AI-assisted diagnosis and treatment: acceptance is substantial but incomplete, with only 3% completely trusting AI and a sizable minority unsure or unwilling. (B) The proportion of respondents who are comfortable with AI-assisted diagnosis, who trust AI (mostly/completely), and who feel positive about AI rises steeply with self-rated understanding of AI in cancer care (adjusted odds ratio roughly 2.5 per level of understanding for comfort with diagnosis, adjusted for age, gender, and ethnicity, *P* < 10^−14^). (C) By age group, older respondents (who make up the majority of pancancer patients) were the most willing to accept AI-assisted diagnosis yet the least likely to report a good or very good understanding of it. AI, artificial intelligence.

Standards, governance, and performant tools address only one side of clinical adoption. The other is whether patients are willing to accept AI as part of their care, and this acceptance cannot be assumed. In an online survey of 475 UK-based members of the public, predominantly current and former cancer patients (85% of respondents), we found that interest in AI was accompanied by substantial caution (Figure 2): only 3% of respondents completely trusted AI to make accurate and unbiased healthcare decisions, 16% did not trust it at all, and approximately one third were unsure about or unwilling to accept AI assistance in their diagnosis (33%) or treatment (36%) (Figure 2A).

The clearest distinction between those willing to accept AI and those more hesitant was not demographic, but educational. Self-reported understanding of AI, rather than age, gender, or ethnicity, was the strongest correlate of acceptance. The proportion of respondents comfortable with AI-assisted diagnosis increased from 32% among those reporting no understanding of AI to 93% among those reporting a good understanding, with similar gradients observed for trust and overall sentiment. This association remained after adjustment for age, gender, and ethnicity, with acceptance increasing by ∼2.5-fold for each level of AI understanding (*P* < 10^−14^; Figure 2B). Higher exposure to consumer AI applications was also associated with increased trust and comfort, suggesting that acceptance is not fixed, but may increase with familiarity and understanding.

This places the oncologist at the centre of responsible AI integration. Older respondents were at once the most willing to accept AI and the least likely to understand it (Figure 2C), leaving the task of closing that gap to the clinician at the bedside. As the trusted professional to whom patients turn when interpreting their care, the oncologist is the natural bridge between AI tools and the people they are intended to serve. However, fulfilling this role requires sufficient AI literacy to explain these tools clearly, contextualise their outputs, and build the understanding on which patient trust depends. Patients prioritise exactly this: three in five respondents (61%) reported that a clear explanation of how AI works would make its use in their care more acceptable.

### Ethical and interpretative oversight of AI tools

Critical for an oncologist will be to maintain ethical and interpretative oversight of the AI tools they are using. As AI becomes increasingly integrated into oncology practice, clinicians need to extend their expertise to encompass new types of clinical tools and approaches. Just as oncologists are expected to clearly communicate the risks, benefits, and mechanisms of action of cancer treatments, they now bear a parallel responsibility to understand and explain the function, outputs, and limitations of AI models used in patient care. A parallel can be drawn with patient-centred pathology clinics, where patients view their assessed tissue and discuss findings with pathologists, improving understanding, trust, and adherence.^25^ Adopting a similar approach in oncology, where clinicians help patients interpret and contextualise AI-derived insights, will foster transparency and confidence in AI-assisted care.

A foundational grasp of how AI models are trained is also essential to identify potential sources of bias that may influence clinical decision making. Moreover, oncologists must maintain oversight of how patient data are utilised in the development and validation of AI tools, and ensure that data are processed within platforms that meet stringent security and governance standards.

**Figure 3:**
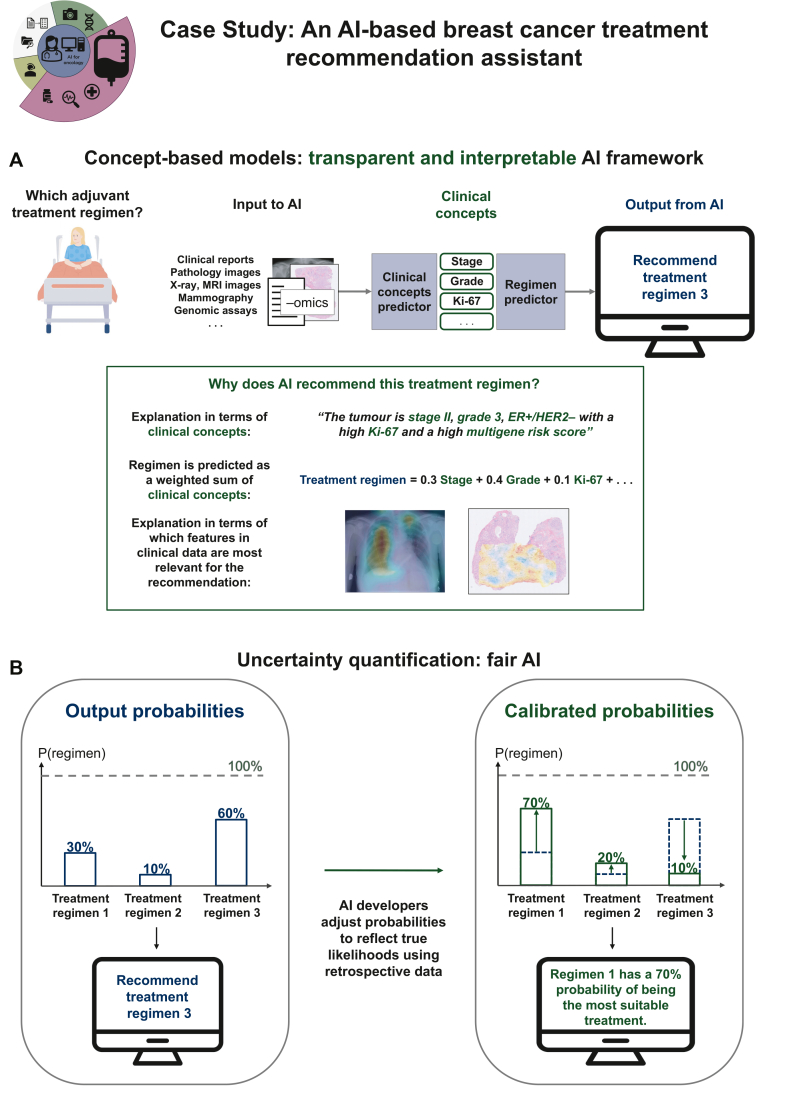
**Conceptual AI model for a clinical decision support system.** This example illustrates an AI tool designed to support adjuvant treatment recommendations for a patient with breast cancer by integrating multimodal data sources (pathology, radiology, molecular data, and clinical reports). The AI assistant is structured around two core principles: (A) Transparent and interpretable AI with concept-based models. Multimodal patient data (clinical reports, histopathology reports and images, medical imaging, molecular data, etc.) are first processed by a concept encoder to predict clinical variables including tumour stage, histological grade, and Ki-67 proliferation index. These predicted concepts form the inputs to a regimen predictor, which combines them to produce a treatment recommendation. This design ensures interpretability and auditability: the oncologist can review, correct, or override predicted concepts, directly inspect how each biomarker contributes to the final recommendation, and visualise which parts of e.g. clinical images were most relevant for the prediction of the clinical variables and treatment recommendation. (B) Fair AI with uncertainty quantification. The AI assistant outputs probabilities. They can be calibrated before deployment into the clinic to reflect real likelihoods of treatment efficacy, and may drastically change the recommended treatment regimen. By making uncertainty quantification explicit, the conceptual AI framework enables bias detection and supports fair and safe decision making. AI, artificial intelligence.

To illustrate these principles, Figure 3 presents a conceptual AI tool designed to recommend adjuvant treatment options for a patient with breast cancer, based on integrated pathology, radiology, molecular data, and clinical reports.

As the number of approved cancer treatments continues to rise (including combinations thereof) and as patients are substratified by an increasing number of biomarkers, AI tools will be able to support clinicians to assess the increasing multimodal diagnostic and clinical data to recommend a patient-specific and time-sensitive treatment regimen. We highlight essential design features of a conceptual AI tool: clinical interpretability and auditability (Figure 3A), fairness across different patient populations (Figure 3B), and secure data handling, illustrating emerging best practices in AI development. Each of these design elements requires consistent monitoring from AI developers and clinicians, as well as robust regulatory frameworks to mandate their responsible integration into AI-assisting tooling, a topic to which we return later in this perspective. These examples are not intended as complete implementation recipes, but as clinician-facing entry points into broader technical and governance requirements, including validation, monitoring, data quality, and deployment. Here, we explore each of these design elements in detail, framed around the three concerns most commonly voiced by patients in our survey: that AI might replace the clinician, that it may be biased or unfair, and that it may fail to safeguard patient data.

### ‘AI will replace the clinician’

One of the most prominent concerns raised by patients in our survey was that AI might displace the clinician rather than support them: roughly one-third of those who described a concern asked, unprompted, that a human professional remain in control of their care, checking and overseeing any AI output, and a further one-sixth feared losing the human touch of cancer care altogether. Patients were consistent on this point, insisting that AI be used ‘in conjunction with, not instead of, human medical professionals’ and that the ‘final decision’ rests with a qualified clinician. The implication is not that patients reject AI, but that they accept it on the condition that an oncologist remains the responsible agent who supervises, interprets and, where necessary, overrides it. The same priority emerged when patients were asked what would make AI more acceptable: assurance that healthcare professionals remain involved was the single most frequently selected option, chosen by more than four in five respondents (84%). This is the human-in-the-loop model of care, and it is what patients are asking for.

For such supervision to be meaningful, however, the oncologist must be able to see and judge how an AI system reached its recommendation. When an AI system ingests clinical data and recommends a treatment path, it may do so without offering any insights into its reasoning, functioning as a ‘black box’. Such opacity makes genuine oversight impossible: without clear insight into the model’s rationale, errors or biases may go unnoticed and inadvertently influence care, and the clinician is reduced to either rubber-stamping or rejecting the output rather than truly supervising it. In the case of a breast cancer patient, the AI model might recommend chemotherapy without indicating whether this decision was based on tumour grade, stage, or genomic pattern, leaving the oncologist unable to vouch for the recommendation to their patient. To be safely integrated into oncology practice, and to keep a clinician genuinely in the loop, AI tools must therefore be held to the same standards of justification and auditability as human experts. This transparency is not only essential for clinical accountability, but also for enabling clear, patient-centred communication about treatment rationale, and thus for sustaining the very human oversight that patients are asking for. Realising this in practice depends on the oncologist being AI-literate enough to interrogate and contextualise these explanations: an uninformed supervisor cannot meaningfully oversee a tool whose reasoning they cannot follow.

In oncology, as in other domains, the development of AI-based tools has predominantly prioritised predictive accuracy over interpretability. Transparency remains limited and is often addressed through *post hoc* explainability tools. Here, we use explainability to refer to attempts to explain a trained model after the fact, and interpretability to refer to approaches in which clinically meaningful structure is built into the model or interface from the outset. In imaging applications, for instance, *post hoc* tools typically highlight the regions of interest deemed most influential to the model’s prediction.^26^^,^^27^ When applied to treatment recommendations based on histopathology images in breast cancer, such tools may emphasise prognostically relevant visual features, such as invasive front or epithelial nests.^28^ However, these fundamentally *post hoc* explanations do not ensure that the model’s decision making is grounded in clinically meaningful or biologically valid concepts.^29^, ^30^, ^31^

Transparency challenges are amplified in multimodal setups.^32^ For instance, if an AI model makes a treatment recommendation for a breast cancer patient, the underlying rationale may be drawn from diverse data sources—radiology, pathology, molecular data, and clinical reports. As a result, oncologists interfacing with a multimodal AI model to support a diagnosis or treatment plan may be shown partial or misleading explanations underpinning model outputs, with limited visibility into how evidence from each modality was weighted or integrated into the final recommendation.

These challenges demand AI models that reason in terms of the same clinical concepts that oncologists use, and that make their decision processes explicit in those concepts, enabling real-time human supervision and potentially enhancing oncologist trust in tools.^33^ For a breast cancer patient, such relevant clinical concepts are standard-of-care biomarkers such as stage, grade, and proliferation index, and for transparency we would like a treatment recommendation to be explicitly grounded in these biomarkers. The AI output, for instance, might read: ‘The recommended treatment regimen for this patient is Treatment Regimen 3 since the tumour is stage II, grade 3, ER+/HER2– with a high Ki-67 and a high multigene risk score’.

A transparent AI model for treatment recommendation could follow a two-step process: Firstly, it would infer clinically relevant features—such as stage, grade, and molecular biomarkers—from the patient’s multimodal data. Secondly, it would generate an interpretable treatment recommendation based on these predicted features. One implementation of this approach is the Concept Bottleneck Model (Figure 3A),^34^, ^35^, ^36^ where the recommendation is presented as a weighted combination of these interpretable clinical biomarkers. These intermediate concepts represent a traceable rationale for the recommendations that oncologists can directly audit, and, when necessary, override. For example, if the model incorrectly predicts a tumour as grade 1, when pathology confirms grade 2, the clinician can correct the input, prompting the model to update its treatment recommendation accordingly.

Similar concept-based approaches have gained broad support within the AI transparency community, demonstrating success across a range of domains, yet their deployment in clinical contexts remains limited.^37^, ^38^, ^39^ We view their adoption in oncology as a decisive step toward a transparent and robust human-in-the-loop integration of AI in cancer health care.

### ‘AI may be biased and unfair’

Alongside the demand for human oversight, the reliability and fairness of AI was among the most common concerns voiced in our survey: roughly a third of patients who described a concern questioned whether AI could be trusted to be accurate and unbiased, citing errors and ‘hallucinations’ in its outputs or the risk that some groups might be treated unfairly (34%). These concerns are well founded: among AI developers, the encoding of biases by AI models is well documented.^40^^,^^41^ A well-known example comes from the US criminal justice system: the COMPAS algorithm, used to inform parole and sentencing decisions, was shown in a landmark ProPublica investigation to systematically propagate racial bias.^42^ In the clinical setting, biases might manifest as misprioritising triage, missing instances of a rare cancer, or skewing eligibility recommendations for a clinical trial.

Biases can enter a model and its predictions at different stages of its development, and an oncologist will need to critically evaluate the potential presence of any biases in any AI tools they are using and understand potential implications on their patients and workflows.

The first potential source of bias lies in the training dataset, particularly how it is sampled from the overarching patient population. An imbalanced training set is likely to result in poor or biased performance for underrepresented patients. For example, studies have shown that skin lesion classifiers (often trained on datasets largely representing light-skinned populations) underperform on darker skin tones and rare lesion types,^43^ while radiology triage models may underprioritise cases originating from certain hospitals or socioeconomic groups.^44^^,^^45^ Similarly, genomics models tuned too narrowly to one cohort can fail catastrophically when applied to others.^46^^,^^47^

The second potential source of bias is how an AI model is designed, as different architectures may prioritise different aspects of the input data. For example, convolutional neural networks (a class of AI models used on imaging data) are often more sensitive to texture than shape,^48^ potentially disadvantaging subgroups whose clinically relevant features manifest differently.

At deployment, biases learned during model development may be amplified when clinical conditions differ from those under which the model was trained and tested. A likely pathway for clinical AI adoption (already emerging across health care systems^49^^,^^50^) is one in which commercial vendors develop AI tools that are subsequently purchased and deployed by healthcare trusts, rather than built in-house. As the scale of data, computational resources, and engineering expertise required for modern AI continues to increase, in-house development is becoming impractical for most institutions. As a result, externally developed AI systems are often trained on patient populations, imaging devices, and acquisition protocols that differ from those of the deploying trust. Such a mismatch typically leads to reduced model accuracy and reliability. This phenomenon, where performance deteriorates because the statistical properties of clinical data change between development and deployment, is commonly referred to as performance drift.

To mitigate these risks, a combination of complementary strategies is required. Firstly, developers should prioritise training AI systems on large, diverse, and representative datasets, and should align model architecture and training strategies with this objective. Secondly, collaboration between vendors and deploying trusts is essential to enable local adaptation of models (such as via fine-tuning) using retrospective data that reflect the trust’s patient population, clinical workflows, and demographics. This local adaptation may be carried out by in-house technical teams or by engineers embedded within the trust in partnership with the vendor.

Nevertheless, it is important to acknowledge that in real-world clinical deployment, some degree of bias is essentially inevitable: no dataset or model can fully capture the clinical, molecular, and phenotypic heterogeneity of patient populations. This reality does not imply that clinical AI is inherently unsafe or ineffective. Rather, it underscores the need for continuous and rigorous monitoring of model bias and fairness throughout deployment.

In principle, major biases can be identified by retrospectively evaluating model errors over time within specific patient subgroups. Disparities in performance relative to the overall population may indicate the presence of bias, and this remains the standard approach for bias assessment. In practice, however, such evaluations are often constrained by the limited availability of reliable clinical ground truth data (for example, what constituted the optimal treatment of a given patient at a particular point in time), especially in complex or multidisciplinary cases. As a result, model performance may be difficult to quantify accurately once a system has been deployed. Moreover, bias detection that occurs only retrospectively carries an inherent ethical limitation: even under ideal circumstances, it implies that some patients have not benefited from the clinical use of the AI system, and in the worst case that certain patients may have been adversely affected by its recommendations.

These challenges point to the need for a paradigm shift in clinical AI, centred on how uncertainty is communicated in real time and at the level of individual patients. AI systems such as the one illustrated in Figure 3A should accompany each recommendation with an explicit estimate of confidence, enabling oncologists to judge the reliability of the output and to integrate it appropriately into clinical decision making.^51^^,^^52^

When model uncertainty is high, AI systems can automatically trigger predefined escalation pathways (such as senior clinician review or additional diagnostic investigations), thereby supporting safer, more informed decision making in oncology practice and strengthening human-in-the-loop workflows. At the population level, persistently elevated uncertainty within specific patient subgroups may indicate the presence of systematic bias. Continuous monitoring of such signals enables developers and deploying institutions to respond proactively, for example by retraining models on more representative data or revisiting model design choices.

To support such workflows, AI systems should move beyond issuing a single recommendation and instead report the estimated probability that each available treatment is the most effective option for an individual patient (for instance, ‘There is a 60% chance the best treatment of this patient is Regimen 3’). However, these probabilities are themselves model-derived estimates and do not automatically correspond to true clinical likelihoods. In particular, models may exhibit overconfidence when confronted with patients whose clinical profiles are rare or insufficiently represented in the training data, producing highly certain recommendations in settings where uncertainty should be high. This behaviour is conceptually analogous to the phenomenon of ‘hallucination’ observed in LLMs trained on incomplete or unrepresentative data, where confident outputs may not be supported by underlying evidence.

From a statistical perspective, it is therefore essential to ensure that reported probabilities and associated uncertainty estimates are aligned with real-world treatment effectiveness. Several robust techniques can be applied before clinical deployment using retrospective data from the deploying site, including probability calibration^53^ (illustrated in Figure 3B), and conformal prediction, which provides stronger safety guarantees.^54^^,^^55^

Collectively, these approaches illustrate some of the range of tools that data scientists and statisticians can offer from their toolbox to help strengthen confidence in AI-assisted cancer care. Ultimately, their clinical value depends on careful integration into governance frameworks and clinical workflows allowing for ongoing monitoring and clinician oversight.

### ‘AI does not safeguard data’

Concern about the security and privacy of personal health data was raised spontaneously by one in five patients in our survey (20%), making it among the most frequently cited worries, with respondents fearing data breaches, unauthorised access, and the sharing or sale of their records to private companies. While many oncologists may not be directly involved in the codesign of AI systems, those who are or those who are approached about contributing their patients’ data to the development of such tools must be assured that these processes adhere to stringent privacy regulations and ethical standards. More broadly, oncologists should feel confident that the AI tools they use safeguard patient data and are developed securely, and be aware of the risks associated with sharing personal health information with external systems. During data collection and model training, the primary challenge lies in the inherently identifiable nature of clinical data. Aggregating such data to train large-scale AI models introduces significant risks of privacy breaches and unauthorised access, underscoring the need for robust safeguards and transparent governance. Safe AI development also depends on data being findable, accessible under appropriate governance, interoperable, and reusable (FAIR), with harmonised metadata, common data models, provenance tracking, and clear data-quality checks.^56^

A promising response at the model training phase is the use of federated learning within secure data environments.^57^, ^58^, ^59^, ^60^, ^61^ In this setup, patient data remain at the local institution, and only model updates or summary outputs, rather than raw data, are shared with a central node and then aggregated. This reduces the risk of patient information leaking beyond institutional boundaries and ensures that institutions retain control over their own data assets. While federated learning is still evolving and requires rigorous governance, it represents a concrete technical solution to part of the security problem. At the same time, federated learning has open challenges that remain the subject of active research, particularly when clinical data are highly heterogeneous. For this reason, uncertainty quantification methods, including those discussed above, require careful local and multisite evaluation before being applied to federated models in clinical settings.^62^ Efforts are underway to put these principles into practice, such as the work of PharosAI,^63^ which is building federated and privacy-preserving infrastructures to enable secure, multi-institutional use of health and biomedical data. Other groups exploring the development of federated solutions in improving data security include, in the UK, the FOCUS-5 (Federating Operations and Collaborations Using the Five Safes) project launched in January 2025,^64^ which builds on the Five Safes framework for providing safe access to sensitive research data^65^ and, in the EU, the Bigpicture community-based platform for pathology images and AI algorithms,^66^ and the German NFDI4health (National Research Data Infrastructure for Personal Health Data) initiative.^67^

A different set of challenges emerges for both oncologists and patients who use pretrained AI models at the point of care. Publicly accessible or individually accessed tools, including general-purpose systems such as ChatGPT,^7^ can be convenient for summarising information or answering clinical questions. However, when they are used outside institutional governance, responsibility is shifted to the individual user. Risks include the entry of sensitive patient data into prompts, opaque or changing model versions and retrieval pipelines, limited local validation, lack of standardised prompts and output templates, incomplete audit trails, and weak institutional oversight.^23^^,^^68^^,^^69^ Such tools should therefore be treated as information aids unless they are deployed within approved clinical governance and data-processing arrangements.

For real-time clinical use, safer deployment models are likely to involve institution-governed architectures: local open-weight LLMs or smaller language models deployed on hospital servers or private cloud infrastructure, or centrally hosted systems provided by technology partners under explicit contractual, technical, and audit controls.^70^ These approaches could support pathway chatbots, ambient AI, LLM-powered clinical scribes, oncology decision-support interfaces, and structured extraction from clinical records for research or trial screening, as illustrated by recent multicentre work using AI to process unstructured lung cancer health records across institutions.^71^ Their advantages include stronger control over patient data, access permissions, model versioning, prompt templates, output formatting, logging, and human review. Their limitations are also substantial: they require investment in secure IT infrastructure, integration with electronic health records, cybersecurity, procurement, clinical validation, model monitoring, and clear accountability for vendor or institutional model updates.

Overall, safeguarding data is not a property of an AI model alone, but of the full deployment architecture around it. The path forward is likely to be driven not only by technology, but also by institutional governance, policy, and regulation. Clinicians should be invited to help shape AI regulation and demand strict compliance from technology companies.

### Regulatory frameworks for responsible AI integration

The introduction of AI tools into the oncology clinic will bring with it yet another set of questions surrounding responsibility and liability. ‘Does ultimate decision-making still fall with the clinical team? Who should be liable if the AI tool makes a prediction that turns out to be suboptimal or if an AI hallucination is acted upon in the clinic? What responsibility should lie with the tool developer or the regulator rather than the oncologist?’ To address these and other questions concerning the transparency and safety of AI, bodies of regulation are now emerging at both national and international levels to govern AI deployment and use in high-stakes domains such as oncology. A notable example is the EU AI Act, in particular Articles 13-15 which mandate transparency, human oversight, and uncertainty quantification.^72^ For AI tools that qualify as medical devices, the EU Medical Device Regulation is also directly relevant, while BS/AAMI 34971:2023 provides AI/machine learning (ML)-specific guidance on applying ISO 14971 risk management to medical technologies that use ML.^73^^,^^74^ In the UK, a new National Commission has just been set up which is designed to support the introduction, this year, of a new regulatory rulebook for accelerating safe access to AI in healthcare and across the NHS.^75^ Regulation, governance, and guidance will need to play a key role in defining protocols for rigorous validation before algorithms are deployed into clinics. This assurance is one that patients actively seek: in our survey, evidence that a tool had been tested and was safe was the second most frequently selected requirement for making AI acceptable, chosen by 70% of respondents. In the United States, the FDA has released a set of principles for Good ML Practice^76^ and in the UK, a new British Standard has been established to evaluate the use of AI within healthcare.^77^ Continuing surveillance of AI systems after deployment will be imperative to ensure patient safety on an ongoing basis. In this light, the appropriateness of the US FDA’s post-market surveillance of AI/ML-based medical devices has been recently reviewed, with suggestions made to strengthen the mechanisms of reporting of adverse events encountered while using such devices.^78^

Until robust regulatory frameworks are developed and implemented, oncologists gain an added responsibility to seek out appropriate practical guidelines and connect with peers who have experience using clinical AI tools. In the UK, for instance, the recent approval by NICE of DERM, a prediagnostic tool to monitor skin health, has prompted the British Association of Dermatologists to create a ‘community of practice’, where clinicians can share experiences and develop guidance for safe use.^79^ Until national regulations mature, oncologists may need to take the lead in forming such communities and setting shared standards to ensure AI is used responsibly in cancer care.

### Preparing oncologists for the integration of AI into clinical practice

As AI technologies continue to evolve, their integration into oncology is inevitable. Preparing both current and future oncologists for this transition requires a structured and practical approach. AI encompasses a broad spectrum of methodologies, and Table 1 outlines key terminology that oncologists are likely to encounter. Many stakeholders have proposed frameworks for the integration of AI education into mainstream oncology training which are supported by high-level interest groups, including the ESMO, the European Interdisciplinary Society of AI for Cancer Research, the American Society of Clinical Oncology, and educator bodies. These programmes must equip clinicians not only with technical and theoretical knowledge but also with an understanding of the ethical, legal, and communication challenges associated with AI use.^80^^,^^81^ At institutions such as Harvard Medical School, generative AI is already being incorporated into medical case discussions with students to help educate them about AI and human collaboration in complex decision making.^82^ Embedding hands-on experience of using generative copilots as interactive clinical decision support systems tools early in training, and a comprehensive discussion around the risks posed such as hallucinations, will be important to ensure oncologists feel comfortable using these tools.

**Table 1:** Definitions of key AI terminology and concepts

Fundamental AI concept	Overview definition
AI	The capacity of computers or other machines to exhibit or simulate intelligent behaviour.
ML	A subset of AI. The capacity of computers to learn and adapt without following explicit instructions, by using algorithms and statistical models to infer from patterns in data.
DL	A subset of ML considered to be in some way more dynamic or complete than others. It is especially used to refer to ML based on artificial neural networks in which multiple layers of processing are used to extract progressively more features from data.
NLP	A form of computational linguistics in which natural language texts are processed by computer (for automatic machine translation, etc.).
LLM	A tool to perform complex NLP tasks. An AI system that processes written prompts and is capable of generating natural language text.
Foundation model	Also known as general-purpose AI, models or systems capable of a range of general tasks.
Narrow scope model	AI models which focus on a specific or limited task, e.g. image classification.
Multimodal model	Models which derive predictions from multiple modalities of data simultaneously (e.g. digital pathology images, text reports, and genetic sequencing data).
Generative AI	AI designed to produce output previously thought to require human intelligence, often in the form of text or images, typically by extrapolating from large collections of data.
Agentic AI	AI systems that are designed to autonomously make decisions and act, able to pursue complex goals with limited supervision.
Robotic process automation	The use of intelligent automation technologies to perform repetitive office tasks, such as filling in forms.
xAI	AI systems that make their decisions understandable to humans, showing the clinical reasoning or evidence behind each prediction.
Concept-based model	Explainable AI systems that make predictions through clinically meaningful concepts (such as tumour grade, receptor status, or genetic subtype) so that their reasoning can be interpreted and validated by clinicians.
Uncertainty quantification	Methods that estimate how confident an AI model is in its predictions, helping clinicians gauge when results are reliable and when they should be treated with caution.
Generalisation	An AI model’s ability to maintain good performance when applied to new, unseen data (such as patients, scanners, or hospitals) different from those it was trained on.
Performance drift	Gradual decline in an AI model’s accuracy or reliability over time as real-world data or clinical practices change from those the model was trained on.
Post-market surveillance	The ongoing monitoring of an AI tool after it has been approved and deployed in clinical practice, to ensure it continues to perform safely, effectively, and as intended.
Hallucination	Information or explanations generated by AI that appear plausible but are actually false or not supported by the underlying data.
Federated learning	Training approach where AI models learn from data distributed across multiple institutions without the data ever leaving its source, protecting patient privacy while enabling collaborative learning.
Ambient AI	AI systems that passively capture and process clinical interactions to automate documentation and reduce administrative burden for clinicians.

AI, artificial intelligence; DL, deep learning; LLM, large language model; ML, machine learning, NLP, natural language processing; xAI, explainable AI.

Training must strike a balance between fostering collaboration with AI and maintaining independent clinical reasoning. This balancing act should be considered both during and after training to mitigate against risks of automation bias—the tendency to overrely on AI systems, potentially overlooking their limitations—and deskilling—the erosion of clinicians’ skills due to overreliance on AI systems.^83^

Given the rapid pace of technological advancement, ongoing education will be vital. Oncologists will benefit from being constantly upskilled through e-learning opportunities or other easily accessible media. Emerging platforms offer flexible e-learning opportunities,^84^^,^^85^ and we envision a future in which oncologists undergo regular assessments of their understanding of AI principles and their clinical implications—similar to external quality assurance schemes used in pathology (e.g. HER2 or PD-L1 scoring).

### Putting patients at the centre in the AI-driven transformation of oncology

In this perspective, we have presented a variety of potential AI applications in oncology, spanning clinical decision support systems, tools designed to relieve the administrative burden on oncologists, and patient support and engagement tools. Using the example of an AI system designed to support breast cancer treatment recommendations, we have explored the key concepts of transparency, bias, and security with which oncologists will need to become familiar, and have discussed emerging methods in the AI field designed to mitigate these risks.

In the future, AI systems in oncology may function similarly to junior clinicians—capable of proposing treatment options, but required to present supporting evidence, reasoning, and confidence levels. This enables senior oncologists to critically evaluate, challenge, or override AI-generated recommendations in consultation with the patient, preserving a human-in-the-loop model that remains firmly patient centred. Currently, the adoption of LLMs in the clinic is constrained by their assignment as general-purpose AI tools, where clinical application requires that, in healthcare, an AI tool must have a specified, narrow scope.^86^ However, the possibility of such systems raises a philosophical question of whether the role of the oncologist may increasingly pivot to one of crafting precise prompts to guide AI models based on LLMs or agentic in nature,^87^ thereby shaping their outputs in complex clinical scenarios.

As AI becomes increasingly embedded in cancer care and research, it is essential that its integration ultimately serves the best interests of patients. Patients must be involved at all phases of the AI lifecycle, spanning tool design, clinical deployment and testing, the development of regulatory frameworks, and the education of oncologists. According to 2022 figures, only 22% of clinical AI tools involved clinicians throughout development; instead, the majority only consulted clinicians once the tool had been developed. The future of oncology will be shaped not solely by technological advancement, but by the collaborative efforts of oncologists and patients. Together, they will guide the development of AI tools that are not only clinically effective but also aligned with patient values and needs. Computer scientists, including ourselves, have a critical role in this process: to provide oncologists with the theoretical foundation and practical skills needed to confidently evaluate, codesign, and implement AI systems. In this highly collaborative framework, oncologists will be empowered to turn technological progress into meaningful innovations with the potential to transform cancer care and outcomes for their patients.
